# Why does apical hypertrophic cardiomyopathy have a favorable outcome than non-apical types?: Cardiac magnetic resonance and echocardiographic findings from 350 patients with hypertrophic cardiomyopathy

**DOI:** 10.1186/1532-429X-16-S1-P233

**Published:** 2014-01-16

**Authors:** Eun Kyoung Kim, Sang-Chol Lee, Hye Bin Gwag, Sung-A Chang, Sung-Ji Park, Sung-Mok Kim, Yeon Hyeon Choe, Seung Woo Park

**Affiliations:** 1 Cardiology, Cardiovascular Imaging Center, Samsung Medical Center, Seoul, Korea, Republic of; 2 Radiology and Center for Imaging Science, Cardiovascular Imaging Center, Samsung Medical Center, Sungkyunkwan University School of Medicine, Seoul, Korea, Republic of

## Background

Apical HCM tends to have a favorable outcome compared to other types. The presence and extent of late gadolinium enhancement (LGE), reflecting myocardial fibrosis, is closely correlated with increased cardiac mortality and strongly associated with surrogates of arrhythmia and subsequent sudden cardiac death. This study sough to investigate the difference in cardiac magnetic resonance (CMR) and echocardiographic and clinical manifestations between apical hypertrophic cardiomyopathy (HCM) and non-apical HCM.

## Methods

A total of 350 consecutive patients diagnosed with HCM (mean age 54 ± 12, 278 males) underwent CMR and echocardiography. Clinical characteristics including risk factors for sudden cardiac death were collected. Eighty-five patients were classified as apical type. On CMR, left ventricle (LV) volumetric parameters were measured, and the amount of LGE was calculated with gray-scale thresholds of 6 SD above the mean signal intensity for normal remote myocardium and also expressed as a ratio against total LV volume. Echocardiographic evaluations included left atrial volume index (LAVI), mitral inflow pattern, tissue Doppler of mitral annulus and LV dimension.

## Results

Patients with apical HCM were less likely to present with history of syncope (2.4% vs. 10.2%, p = 0.02) and have family history of sudden cardiac death than those with non-apical HCM (5.9% vs. 15.8%, p = 0.02). Functional class was also more favorable in apical HCM (frequency of NYHA class I; 89.4% vs. 66.8%, p < 0.001). CMR volumetric parameters were not different between the two groups except LV mass index (71.7 ± 17.3 vs. 92.0 ± 34.1, p < 0.001). LGE was less frequently detected (87.1% vs. 93.9%, p = 0.04), and the amount of LGE was significantly smaller in apical HCM (7.0 ± 6.0% vs. 14.6 ± 10.5%, p < 0.001). The E/e level and LAVI were also lower in apical HCM patients (E/e; 10.1 ± 3.3 vs. 13.6 ± 5.7, p < 0.001 and LAVI; 40.4 ± 20.8 vs. 45.9 ± 16.9, p < 0.001).

## Conclusions

Apical hypertrophy shows relatively small burden of myocardial fibrosis and less severe diastolic dysfunction, and subsequently more favorable clinical manifestations in comparison with other HCMs. This may be one explanation of why most patients with apical HCM show a benign course of disease compared to non-apical HCM.

## Funding

None.

